# Management of an Intracanal Separated Instrument: A Case Report

**Published:** 2013-10-07

**Authors:** Dipti Choksi, Barkha Idnani, Devendra Kalaria, Ronak N. Patel

**Affiliations:** a Department of Conservative Dentistry and Endodontics, DDU, Nadiad, Gujarat, India;; b Reader Conservative Dentistry and Endodontics, RAU Dental College and Research Center, Indor M.P. India

**Keywords:** Calcified Canal, Endodontics, Fractured Instrument, Masserann Kit

## Abstract

Instrument fracture within the root canal during root canal treatment is an unwanted and frustrating complication. The fractured segment may hinder cleaning and shaping procedures with potential impact on prognosis of treatment. Fracture of endodontic instrument often results from incorrect use or overuse. If breakage occurs clinically, the patient should be informed of the incident and consideration should be given whether to remove the fragment or not. When managed properly, the presence of a broken fragment per se may not adversely affect the outcome of root canal treatment. This article reports management of an intracanal separated instrument. Masserann kit along with gates glidden drills were used to remove the intracanal broken instrument.

## 1. Introduction

Every clinician who has performed endodontics has experienced a variety of emotions ranging from the *thrill of the fill* to an upset, like the procedural accidents such as intra canal separation of an instrument. During root canal preparation procedures, the potential for instrument breakage is always present. When instrument breakage occurs, it immediately provokes despair, anxiety, and then the hope that nonsurgical retreatment techniques still exist to liberate the instrument from the canal [1].

Most of the stainless steel instruments fail by excessive torque and NiTi rotary files usually fracture because of torsional stress and cyclic loading. Fractured instrument itself may not cause treatment failure. However, the remaining fragment in the root canal can hinder proper preparation of root canal space. Masserann kit is one of many devices that have been proposed for removal of the fractured fragment [2]. In presence of separated instrument the outcome is affected. Fox et al. also concluded that failed cases were associated with intracanal broken instruments [3]. Broken separated instrument when retained might produce corrosion products in the canal and thereby leads to the endodontic failure [4]. The following case describes the clinical scenario of a separated intracanal instrument removal by means of Masserann kit.

## 2. Case Report

A 45 year old female was referred to us with a separated endodontic instrument in the calcified root canal of maxillary right lateral incisor [3]. An intentional root canal therapy was advised as a part of treatment plan for full mouth rehabilitation. During which the procedural accident had happened.

**Figure 1.**
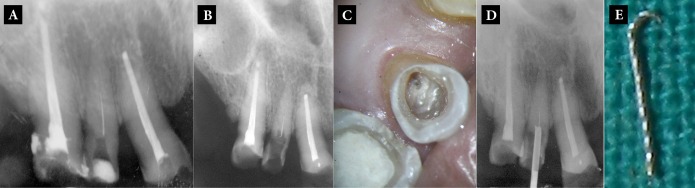
A) Periapical radiograph: Separated instrument is visible in middle 3rd of calcified root canal in maxillary right lateral incisor; B and C) Making a channel around the separated instrument to keep the broken instrument in the center of the tube of Masserann Kit; D and E) Engaging tube of Masserann Kit with the separated instrument and removal of the fragment from root canal

On clinical examination, generalized tooth abrasion was found. Vitality test of tooth #12 revealed no response. On radiographic examination, a separated endodontic instrument was found in middle third of the root canal. The canal was also calcified and narrow (Figure 1A). Patient was informed about the instrument separation and removal of the fragment was chosen as the treatment plan. 

Masserann micro kit (Micro-Mega, Besanc¸on, France) was used to retrieve the instrument. Initially a gates glidden drill and ultrasonic Kerr K-file tips (#15-20) were used to keep the broken instrument in its center and to cut a circumferential trough around the fragment (Figures 1B and 1C). The metal tube was then fitted over the 'freed' end of the fragment, to engage it by means of a central stylus that was screwed in position. The instrument was then removed from the canal (Figures 1D and 1E). 

**Figure 2.**
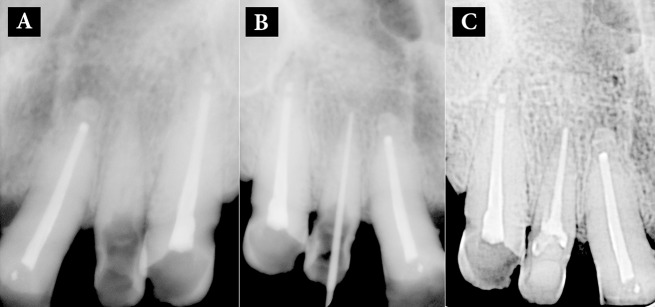
A) After removal of separated instrument; B) Negotiation of full working length of calcified root canal, and C) Obturation

Root canal treatment was performed (Figures 2A-C). Working length was established using a size 15 K-file (Mani, Huaxian, China). Root canal irrigation was done using warm 3% NaOCl solution and then 17% EDTA was used to fully negotiate the narrow, calcified root canal. Canal was prepared using hand up to size 45. Obturation was done using 2% gutta-percha cones with lateral condensation technique. Access cavity was then sealed using hybrid composite (3M ESPE, St. Paul, USA). Early treatment goals were achieved without complication and patient was referred to prosthodontic department for final restoration. 

## 3. Discussion

Procedural errors in endodontics can occur during the process of root canal treatment that can be a result of factors over which the operator may or may not have control. Stainless steel instruments usually fail by excessive amounts of torque and NiTi instruments break due to combined action of torsional stress and cyclic loading. Factors affecting failures are instrumentation technique, use of torque controlled motor, core dimension and surface conditioning of the instrument, rotation rate, radius of canal curvature, presence of straight line access and glide path to apical portion of the canal. Root canal instruments are indispensable for root canal space preparation. An instrument can fracture if its ultimate strength is exceeded, or when a crack has propagated to such a degree that the remaining cross section of the instrument is unable to bear the operating load. Smaller endodontic instruments (size 15, 20) are more prone to distortion as a result of stressing on their small cross sections. Fractured fragment itself may not cause treatment failure but its being stock within the root canal can prevent improper preparation and disinfection resulting in a negative effect on the treatment outcome [2]. Attempts to remove fractured instruments can lead to ledge formation, over enlargement and transportation of prepared root canal or can lead to perforation. Hence the clinician has to evaluate the options of attempting to remove the instrument, bypassing it or leave the fractured fragment in the canal. The decision making should be made with the consideration for pulp status, canal infection, canal anatomy, position of the fragment and the type of fractured instrument [5-7]. Most commonly used devices to remove the fractured instruments are: ultrasonic devices, extraction tubes (Masserann kit), Canal Finder system and manual instruments. The main determinant for removal of the fractured fragment is the location of the fragment in relation to the curvature of the root canal. If the fragment is situated coronal to the curve, removal of the fragment is possible; on the other hand if the separation occurs beyond the curvature the retrieval is deemed impossible. Removal of fractured fragment from the root canal requires manual skills, equipments, instruments and good knowledge of root canal anatomy [2, 8]. Recommended guidelines to retrieve the instruments are:

1.Obtain a visual access of the coronal end of the fragment;

2.Knowledge about the root canal anatomy;

3.Attempt to bypass the fragment at first stage;

4.Choosing the right armamentarium [2, 9, 10].

To prevent the unpleasant mishap of instrument separation from happening, proper measures have to be taken during the treatment, such as; 

1.Whenever possible, straight-line access to the apical portion of the canal should be created; 

2.Glide path to the working length has to be established using #10 and #15;

3.Recommended torque control motor has to be used for particular instruments;

4.The file has to be advanced slowly and gradually in the canal until the resistance is felt;

5.Use of rotary files in abruptly curved canals has to be avoided;

6.Greater safety margins have to be allowed for instruments used in conjunction with NaOCl due to detrimental effects of corrosion; and

7.Smaller instruments are prone to fracture hence they are recommended to be single used [2, 6, 11, 12]. Curved and narrow canals have a higher risk of instrument fracture than straight and wide canals.

Since most stainless steel instruments fracture with excessive amount of torque, care has to be taken during negotiation and instrumentation of narrow, curved root canals. Various techniques and treatment modalities are available for instrument retrieval form root canal. This article describes a case of management of instrument separation by the use of Masserann kit [13].
